# Quality improvement in juvenile idiopathic arthritis: a mixed-methods implementation pilot of the CAPTURE-JIA dataset

**DOI:** 10.1186/s12969-022-00697-4

**Published:** 2022-06-18

**Authors:** Flora McErlane, Chris Anderson, Saskia Lawson-Tovey, Barbara Lee, Chris Lee, Laura Lunt, Janet E. McDonagh, Andrew D. Smith, Nicola Smith, Gavin Cleary

**Affiliations:** 1 Paediatric Rheumatology, Great North Children’s Hospital, Newcastle Hospitals NHS Foundation Trust, Newcastle upon Tyne, UK; 2 Institute of Population and Health Sciences, Medical School, Newcastle University, Newcastle upon Tyne, UK; 3 Appligo Ltd, https://www.agileware.io/about; 4 Centre for Genetics and Genomics Versus Arthritis, Centre for Musculoskeletal Research, The University of Manchester, Manchester, UK; 5 National Institute of Health Research Manchester Biomedical Research Centre, Manchester Academic Health Science Centre, Manchester University NHS Foundation Trust, Manchester, UK; 6 Versus Arthritis Centre for Epidemiology, Centre for Musculoskeletal Research, The University of Manchester, Manchester, UK; 7 Department of Paediatric and Adolescent Rheumatology, Royal Manchester Children’s Hospital, Manchester University Hospitals NHS Trust, Manchester, UK; 8 Musculoskeletal Research Group, Translational and Clinical Research Institute, Newcastle University, Newcastle Upon Tyne, UK; 9 Department of Rheumatology, Alder Hey Children’s Hospital, Liverpool, UK

**Keywords:** Juvenile idiopathic arthritis, Quality improvement, Electronic data collection, Paediatric rheumatology

## Abstract

**Background:**

A significant proportion of children and young people with juvenile idiopathic arthritis (JIA) do not achieve inactive disease during the first two years following diagnosis. Refinements to clinical care pathways have the potential to improve clinical outcomes but a lack of consistent and contemporaneous clinical data presently precludes standard setting and implementation of meaningful quality improvement programmes.

This study was the first to pilot clinical data collection and analysis using the CAPTURE-JIA dataset, and to explore patient and clinician-reported feasibility and acceptability data.

**Methods:**

A multiphase mixed-methods approach enabled prospective collection of quantitative data to examine the feasibility and efficacy of dataset collection and of qualitative data informing the context and processes of implementation. An initial paper pilot informed the design of a bespoke electronic data collection system (the Agileware system), with a subsequent electronic pilot informing the final CAPTURE-JIA data collection tool.

**Results:**

Paper collection of patient data was feasible but time-consuming in the clinical setting. Phase 1 paper pilot data (121 patients) identified three themes: problematic data items (14/62 data items received >40% missing data), formatting of data collection forms and a clinician-highlighted need for digital data collection, informing Phase 2 electronic data collection tool development. Patients and families were universally supportive of the collection and analysis of anonymised patient data to inform clinical care. No apparent preference for paper / electronic data collection was reported by families.

Phase 3 electronic pilot data (38 patients) appeared complete and the system reported to be easy to use. Analysis of the study dataset and a dummy longitudinal dataset confirmed that all eleven JIA national audit questions can be answered using the electronic system.

**Conclusions:**

Multicentre CAPTURE-JIA data collection is feasible and acceptable, with a bespoke data collection system highlighted as the most satisfactory solution. The study is informing ongoing work towards a streamlined and flexible national paediatric data collection system to drive quality improvement in clinical care.

**Supplementary Information:**

The online version contains supplementary material available at 10.1186/s12969-022-00697-4.

## Background

The term juvenile idiopathic arthritis (JIA) encapsulates the internationally recognised classification system for the chronic childhood onset arthritides [1, 2]. JIA is a heterogeneous group of diseases with wide variation between International League for Arthritis and Rheumatism (ILAR) subtypes [3]. Although JIA is one of the most common chronic inflammatory diseases of childhood, numbers of new patients presenting to individual centres, particularly those with less common ILAR subtypes, are too low for meaningful analyses of local datasets [2].

.As a consequence, the majority of data informing our understanding of clinical outcomes in JIA are derived from clinical research. Early aggressive therapy has been shown to result in relatively high numbers of children and young people (CYP) with polyarticular JIA achieving clinically inactive disease by 6 months [4, 5]. However, a number of multicentre observational studies have demonstrated that a significant proportion of CYP do not achieve inactive disease within the first 1-2 years of routine clinical care [6, 7]. Achievement of inactive disease is associated with disease subtype and is less likely in the presence of diagnostic delay [8, 9], suggesting that improvements in the quality and consistency of clinical care have the potential to significantly impact clinical outcomes.

Traditional clinical studies and clinical trials are highly expensive and recruitment of a representative spectrum of CYP with JIA can be challenging. Furthermore, the formal rigidity of clinical trial data collection is not representative of routine clinical care and can be difficult to sustain over long periods of time [10]. Harmonisation of research-quality clinical data collection with routine clinical care would enable multicentre analyses, facilitating collaborative and effective working, enabling benchmarking of clinical services against quality indicators and aligning treatment strategies and clinical research opportunities.

With this in mind, the Canadian Alliance of Pediatric Rheumatology Investigators (CAPRI) developed a non-traditional, minimal data-collection JIA Registry [10]. Initial findings suggest that treatments for JIA in Canada have intensified, with 81% of patients attaining inactive disease within one year of diagnosis.

In the UK, the first National Clinical Audit for Rheumatoid and Early Inflammatory Arthritis (NCAREIA) was launched in 2014, comprehensively benchmarking clinical care in newly diagnosed inflammatory arthritis presenting over the age of sixteen years. The widespread variation in clinical care identified by the audit led to development of the National Early Inflammatory Arthritis Audit (NEIAA), commissioned by the Healthcare Quality Improvement Partnership.

The first NEIAA Annual Report (May 2018 to May 2019) assessed quality of care against seven key metrics, identifying frequent delays in referrals from primary care and significant variations in departmental staffing ratios [11]. The data enabled services to open a dialogue with commissioners and, in certain cases, secure service improvements by means such as reconfiguration and additional staffing. The second annual report (May 2019 to May 2020) demonstrated significant improvements in time from referral to first specialist appointment and treatment initiation [12].

The NEIAA has demonstrated that sustained collection of key clinical data items is possible in the UK and precedes important improvements in quality of clinical care. Furthermore, active participation in the national audit programme correlates with the quality of care provided [13]. There is therefore a pressing need to develop a robust national data collection system for childhood-onset arthritis, enabling implementation of important quality improvement work benchmarking, standardising and improving paediatric and adolescent clinical care.

The collaborative and methodologically robust development of CAPTURE-JIA, an agreed, clinically relevant quality-of-care ‘core dataset’ for JIA, has been reported previously [14]. Designed to collect complete information at each visit relevant to disease outcomes, service delivery and research, it includes those data items previously identified as necessary for national clinical audit together with novel JIA-specific patient-reported outcome and experience measures (PROM and PREM) developed and validated in association with the UK patient and parent community [15, 16]. The CAPTURE JIA dataset is summarised in Table 1.

**Table 1:** The CAPTURE-JIA quality of care dataset

Data Category	Data Item (***N*** = 62)	Visit
Demographic data	1.1 NHS number of patient	All
	1.2 Date of attendance / visit date	All
	2.1 Gender	First
	2.2 Date of birth	First
	2.3 Ethnicity	First
Clinical history data (diagnosis and disease features)	6.1 Date of symptom onset	First
	6.2 ILAR sub-type	First/Clinically indicated
	6.3 Date of diagnosis	First/Clinically indicated
	6.4 Relevant co-morbidities	First/Clinically indicated
	6.5 Morning stiffness of joints	All
	6.7.A Systemic features	First/Clinically indicated
	6.7.B Systemic JIA Global Assessment	First/Clinically indicated
	6.8.A Uveitis history	All
	6.8.B Uveitis status at most recent eye examination	All
Medication data	7.1 Medication name	All
	7.2.A Date of decision to treat or change treatment	All
	7.2.B Date treatment started / date of single treatment	All
	7.3 Dose	All
	7.4 Frequency	All
	7.5 Route	All
	7.6 Date medication stopped or changed	All
	7.7 Reason for stopping or changing medication	All
	7.8 Joints injected with intra-articular steroids	All
	7.9 Adverse drug reactions	All
Examination data	3.1 Height	All
	3.2 Weight	All
	6.6 Leg length discrepancy	First/Clinically indicated
COV / patient reported data	4.1.A Active joint assessment	All
	4.1.B Swollen joint assessment	All
	4.1.C Tender joint assessment	All
	4.2 Limited joint assessment	All
	4.3 Physician Global Assessment	All
	4.4 Patient / Parent Global Assessment of overall well-being	All
	4.5.A CHAQ / HAQ (final numeric score)	All
	4.5.B CHAQ multiple choice questions	All
	4.5.C CHAQ yes/no questions	All
	8.10 Date form completed (CHAQ/PREM/PROM)	All
	8.11 Form type (patient or parent) (CHAQ/PREM/PROM)	All
	8.12 Completed by (CHAQ/PREM/PROM)	All
	8.13 Time taken to complete form (CHAQ/PREM/PROM)	All
	8.14 JIA-specific patient reported experience measure (PREM)	All
	8.15 JIA-specific patient reported outcome measure (PROM)	All
Once only lab data	5.1 RF +/−	First/Clinically indicated
	5.2 HLA B27 +/−	Once if indicated
	5.3 ANA +/−	First/Clinically indicated
	Structure of the care team	
Quality of care data	8.1 Date of referral letter being received in rheumatology department	First
	8.2.A Date of first appointment offered in a rheumatology clinic	First
	8.2.B Date of first appointment in a rheumatology clinic	
	8.3 Does steroid injection specify a dedicated Paediatric GA list?	All
	8.4 Date of first eye screen	All
	8.5 Date patient was counselled before starting methotrexate	All
	8.6 Date patient was counselled before starting a biologic	All
	8.7 Clinic type / organisation	All
	8.8.A Is patient eligible for recruitment to BSPAR Etanercept Study?	All
	8.8.B Has patient been recruited to the BSPAR Etanercept Study?	All
	8.9.A Is the patient eligible for recruitment to the BCRD study?	All
	8.9.B Has the patient been recruited to the BCRD study?	All

This paper reports the first multisite pilot of CAPTURE-JIA clinical data collection.

## Aims

The aims of this study were to pilot CAPTURE-JIA data collection and analysis across multiple UK paediatric rheumatology centres and collect patient and clinician reported feasibility and acceptability data in order to (i) determine the feasibility of multisite data collection (ii) identify an effective data collection system and (iii) understand the acceptability of data collection to families and to clinical teams.

## Methods

**Fig. 1:**
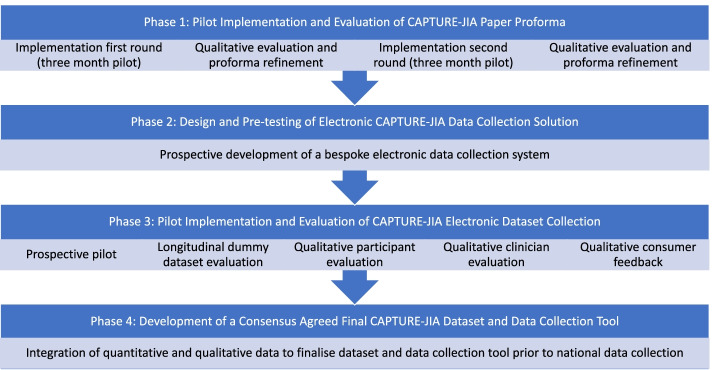
Flow chart summarising methodologies used

We employed a multiphase mixed methods approach (convergent study design), using quantitative data to examine the feasibility and efficacy of dataset collection and qualitative data to better understand the context and processes of implementation (Fig. 1). Datasets were merged to identify key themes and draw conclusions about optimal data collection processes.

The study complies with the Declaration of Helsinki. The locally appointed ethics committee approved the research protocol [National Research Ethics Committee East Midlands-Leicester IRAS 212656]. Detailed study information was provided and discussed prior to taking written consent and assent (if applicable). Participation was voluntary and patients/parents were made aware that participation would have no impact on the care they or their child receive(s). There were no physical or psychological risks to participants taking part.

### Phase 1 methodology: pilot implementation and evaluation of a CAPTURE-JIA Paper Proforma

A purposive sample of six tertiary Paediatric Rheumatology centres was invited to collect the CAPTURE-JIA dataset using paper collection forms at consecutive clinics to a total of twenty patients per centre. Six virtual clinician focus groups (*n* = 3-10 participants) were conducted by an experienced qualitative research assistant (NS) based at Newcastle University (post PhD). Think aloud techniques were used to elicit clinicians’ views on acceptability and feasibility, informed further amendments to the data collection tools and data dictionary. A second implementation pilot preceded a further qualitative evaluation and proforma refinement.

### Phase 2 methodology: design and pre-testing of an electronic CAPTURE-JIA data collection solution

The need for an electronic data collection system was identified prior to the study and further highlighted by Phase 1 clinician feedback. During Phase 2, the study team worked with a company called Appligo Ltd., (https://www.agileware.io/about) experienced in development of modern and bespoke cloud-based data collection systems, to develop a purpose-built electronic platform to collect and store the CAPTURE-JIA dataset. The platform is stand-alone, and data are stored securely. Access is over the internet using any modern, HTML5 compatible browser and is not restricted to a specific operating system or device. The platform was designed to link with (and/or pull data from) local electronic patient record (EPR) systems or may be used in parallel with existing hospital systems. Data may be collected in the clinical setting, using desktop computers, laptops, iPads, or smartphones. The Appligo senior team have considerable experience of delivering NHS healthcare projects as described in Supplemental Material S1.

The Agileware “environment” was set up at the University of Manchester. WT and team supported the initial design of the screen. Each pilot site had its own domain. Participants were allocated a study ID with patient identifiable data stored securely within the local Trust only. Desensitised data were received at University of Manchester.

In accordance with recognised Quality Improvement methodologies, the Agileware solution was pre-tested at two UK Paediatric Rheumatology centres (Newcastle Hospitals and Alder Hey Hospital) and exhibited at the British Society for Paediatric and Adolescent Rheumatology (BSPAR) 2019 Conference, with the aim of identifying potential barriers and challenges to national implementation and collecting live feedback from clinical users.

### Phase 3 methodology: pilot implementation and evaluation of CAPTURE-JIA electronic dataset collection

Phase 3 aimed to establish whether multi-site electronic collection of a complete and analysable CAPTURE-JIA dataset is feasible. This phase of the study included 4 workstreams:

A) Prospective electronic pilot:

The purpose-built electronic platform was used to collect a pseudonymised-CAPTURE JIA dataset from up to 60 CYP with JIA attending one of 3 original pilot sites (Newcastle Hospitals, Alder Hey and Manchester Children’s Hospital; *N* = 20 per site). Clinicians recorded feedback on the electronic data collection tool at the end of each data collection clinic.

B) Longitudinal dummy data analysis:

A dummy longitudinal dataset was entered into Agileware exploring whether longitudinal data can be extracted and analysed meaningfully. We assessed the extent of manipulation required to analyse the data and tested whether the refined data could answer the national audit questions defined in the JIA National Audit Methodology paper [15]. Extracted data were stored in accordance with the Data Protection Act (2018) and the University of Manchester Information Security and Data Protection Policies on a strictly controlled data storage area within the University’s network infrastructure with regular back up.

III) Qualitative participant evaluation:

A feedback questionnaire exploring the most user-friendly way to collect patient-reported CAPTURE-JIA data items was developed and pre-tested by an experienced qualitative researcher (NS). No changes were made prior to administration to consented families.

IV) Qualitative clinician evaluation

A virtual clinician focus group was facilitated by an experienced clinician researcher (FM); the group included her direct clinical colleagues as well as clinicians from other tertiary centres. Pilot clinician feedback was reviewed and discussed with the group encouraged to suggest challenges and review potential solutions in more depth.

### Phase 4 methodology: development of a consensus agreed final CAPTURE-JIA dataset and data collection tool

Participant, clinician, and consumer feedback were used to inform any necessary changes to the CAPTURE-JIA dataset and the electronic data collection solution.

Following completion of the study, the national youth research advisory panel (Your Rheum - https://yourrheum.org) - a UK-wide young persons’ advisory group for young people aged 11-24 years interested in adolescent and young adult rheumatology research - provided additional insights into the patient data collection methodologies.

### Data analysis

Quantitative CAPTURE-JIA pilot data were analysed using descriptive statistics with qualitative techniques applied to any free-text comments. Questionnaire and focus group data were analysed qualitatively in accordance with standard procedures of rigorous qualitative analysis [17]. Procedures from first-generation grounded theory (coding, constant comparison, memoing) [18], analytic induction (deviant case analysis) [19] and constructionist grounded theory (mapping) were used [20]. Data collection and analysis occurred concurrently so that issues raised in earlier rounds were explored subsequently. We undertook independent coding and cross checking, and a proportion of data was analysed collectively in ‘data clinics’ where the research team shared and exchanged interpretations and key issues emerging from the data.

## Results

### Phase 1

One hundred and twenty-one patients were recruited over three months. The completeness of the dataset was similar across centres, with minor variations. The majority of data items were > 80% complete. However, 14/62 data items received >40% missing data. (Table 2) Further descriptive analyses highlighted incorrect completion of paper forms.

**Table 2:** CAPTURE-JIA data items with >40% missing data in paper pilot

Data item	% missing (if item required)
Relevant co-morbidities?	60
Macrophage activation syndrome?	100
Has the ILAR subtype changed since previous visit?	50
Morning stiffness lasting >15 minutes	42
History of any form of uveitis?	52
Date started uveitis medication?	50
Uveitis medication details	83
Counselled prior to new disease-modifying drug (DMARD) / biologic?	56
Enrolled in national biologic registries if new DMARD / biologic?	48
Joint count (homunculus or table format)	48
Physician assessment of systemic disease activity (VAS)	75
Erythrocyte sedimentation rate (ESR)	74
C reactive protein (CRP)	92
Plasma viscosity	100

Three themes emerged from the focus groups: problematic data items (defined as >10% missing at >1 centre), format of clinician data forms and the role of digital data collection. Suggested solutions included minor changes to data item definitions and formatting. There were no refinements to the data items. Development of a digital data collection system was identified by all as essential.

Due to a lack of clear consensus, the original forms included a number of ways to record joint count data. This proved confusing and a unanimous decision was taken to collect joint count data on all 83 joints in a tabular format.

### Phase 2

The Agileware solution was exhibited at a national UK paediatric rheumatology conference (BSPAR 2019) where delegates visiting the exhibit were asked to complete a short survey (Table 3). 93% of responses indicated that Agileware was either *Very Easy* or *Easy* to use, suggesting minimal training would be required. NHS providers are at varying degrees of technical and transformational change, resulting in a mixed economy of patient data collection methodologies. A recurring theme across all responses was the limited resource / capacity with internal IT departments with an associated reluctance to take on more work and integrate external systems. Proposed solutions included: communication / discussions with individual IT departments, a national directive (for example mandated national audit) and local/national funding opportunities.

**Table 3:** Agileware solution clinician feedback BSPAR 2019

Question	Responses	
On a scale of 1-5 how easy did you find using Agileware?	Very easy	57%
	Easy	36%
	Neutral	7%
	Difficult	–
	Very difficult	–
On a scale of 1-5 how enthusiastic are you to use the Agileware system to collect the CAPTURE JIA dataset for JIA patients?	I would really like to	79%
	I would like to	21%
	I have no strong feelings	–
	I have little interest	–
	I have no interest	–
On a scale of 1-5 how likely is the adoption of Agileware at your hospital?	Very likely	36%
	Likely	21%
	Neither likely nor unlikely	36%
	Unlikely	7%
	Very unlikely	–
What is your local Electronic Patient Record (EPR) maturity?	EPR currently used in practice	54%
	EPR in development	23%
	Plans for future development of EPR	15%
	No plans for EPR	–
	Don’t know	8%
If patient data are collected electronically: what method do you use?	EPR	56%
	Dedicated database	31%
	Excel spreadsheet	13%

### Phase 3

#### Prospective electronic pilot

COVID-19 restrictions on ongoing NHS research and reduced staff availability resulted in recruitment from just two of the three study centres; seven paediatric rheumatology consultants and one paediatric rheumatology grid trainee participated in the electronic pilot with 38 patients recruited across Alder Hey and Newcastle Hospitals.

Data entry was reported to be neutral to easy overall, with clinicians reported completion times ranging from 11 to 30 min (average 20.7 min per form). There was a clear trajectory towards improved confidence and faster data entry with experience using the system. Data entry was significantly faster for new JIA patients; clinicians reported time-consuming delays associated with the identification of historical clinical data. Clinicians were positive about the system in a post-pilot clinician focus group, reporting that the system looks good, is easy to navigate and flows in accordance with the clinical consultation. There was universal agreement that the system is more user-friendly than paper data collection and the ability to review summary data at local hospital level was viewed as an important advantage.

It was not possible to directly export data from either hospital EPR to the Agileware system. Although the Agileware system houses data in accordance with the Data Protection and Security Toolkit (DPST) provided by NHS Digital, neither Trust had systems in place to allow new direct data exports during the study recruitment period.

Although the electronic forms were all complete, the number is too small to provide robust insight into data completeness and would not accurately represent reasons for missingness in a standard clinic setting at an overall and individual site level.

#### Longitudinal dummy data entry and analysis

A dummy dataset of 20 patients with linked data entries over multiple time points was developed by participating clinicians. Data were analysed in combination with the prospective electronic pilot data to ascertain whether the HQIP National Audit questions were answerable [15].

.Seven of the eleven audit questions could be answered within the Agileware system itself. A number of graphical additions to the database, namely percentages, ensured that the 7 audit questions can be built in individual site environments, allowing each paediatric rheumatology centre to independently monitor performance.

The remaining questions were not answerable within the Agileware system; they had to be extracted and analysed using a statistical package (Stata version 14.0). Once extracted, it was relatively simple to transform the data and produce answers to the audit questions. Due to the small dummy dataset, some more specific questions were not fully answerable, though we were able to generate proof of concept that they could be answered with a larger dataset.

Audit question analysis methodologies presented in Table 4.

**Table 4:** HQIP national audit question analysis

Subject area		Proposed Question	Answerable within Agileware	Answerable within a statistical package
1. Categorisation	1A	What is the number of patients in each ILAR sub-group in the audit population?	Yes	Yes
	1B	What is the proportion of patients in each ILAR sub-group, relative to the audit population?	Yes	Yes
2. Access	2	What is the median time for children with suspected JIA, from receipt of the referral letter in the Rheumatology department to the date of the first appointment offered in a rheumatology clinic? (modified PRH03)	Yes	Yes
		(PRH03: children with newly diagnosed JIA should have access to a specialist paediatric rheumatology service* within 6 weeks of the referral being received by the specialist service)		
3. Steroids	3A	What is the mean number of days to joint injection on a dedicated Paediatric GA list from date of decision to treat, for children of different ILAR sub-types? (PRH04)	Yes	Yes
		*(PRH04: Children with JIA who need to have intra-articular steroid injection(s) should wait no longer than 4 weeks for the procedure. Those needing general anaesthesia (GA) will have these performed on a Paediatric GA list.)*		
	3B	What percentage of children of different ILAR sub-types is on oral (systemic) steroids at different times after their first Rheumatology clinic visit?	No	Yes
4. DMARDS	4	What is the median time from their first clinic visit to the decision to treat with methotrexate, for children of different ILAR sub-types?	No	Yes
5. Biologic therapies	5	What is the median time from their first clinic visit to the decision to treat with their first biologic therapy:	No	Yes
		- for children of different ILAR sub-types?		
		- for different biologic therapies?		
6 Uveitis	6	What is the median time from the patient’s first clinic visit to the date of their first uveitis screening with an appropriate paediatric ophthalmic specialist, for patients of different ILAR sub-types? (modified PRH05)	No	Yes
		*(PRH05: Children with Juvenile Idiopathic Arthritis should have access to Uveitis screening within 6 weeks of diagnosis)*		
7. Clinic organisation	7A	What proportion of children who started a DMARD or biologic agent were counselled by a Paediatric Rheumatology Clinical Nurse Specialist (PRH01)	Yes	Yes
		(PRH01: Children with established rheumatic diseases (and their carers) should be counselled by a Paediatric Rheumatology Clinical Nurse Specialist* before starting treatment with a DMARD or Biologic.)		
	7B	What proportion of children with JIA is seen in a specialist paediatric rheumatology clinic and what proportions in other clinic types (modified PRH02)	Yes	Yes
		(PRH02: Children with Juvenile Idiopathic Arthritis (JIA) should have access to a paediatric rheumatology clinic* for follow-up appointments)		
8. Research	8	What proportion of eligible patients has been recruited to the BSPAR Cohort Studies (BSPAR Etanercept and BCRD)?	Yes	Yes

#### Qualitative participant evaluation

A total of 40 CYP/parent pairs completed patient-data collection preference questionnaires (120 questionnaires received in total, one for each form type, e.g. CHAQ, PROM and PREM).

CYP / parent pairs were happy to complete the forms although there was a definite lack of consensus relating to paper or electronic formatting. Overall, 61/120 (50.8%) pairs highlighted a preference for paper forms with 48/120 (40%) specifying a preference for electronic forms.

Electronic forms, completed at home, were highest ranking for both the CHAQ (*n* = 15/40) and PROM (*n* = 12/40) but electronic and paper versions of the PREM (*n* = 11/40 respectively), completed either at home or in the hospital waiting area, were ranked equally highly. If all forms were to be completed electronically, accessing the forms via personal mobile phone was favoured (*n* = 87/120) over personal iPad/tablet (*n* = 59/120) or hospital iPad/tablet (*n* = 56/120). Nearly half of respondents would prefer a text reminder to complete the forms prior to the appointment (*n* = 54/120), followed by email (*n* = 27/140) or letter (*n* = 36/120).

Completion of forms between appointments was equally divisive. Around 30% respondents would choose to complete the CHAQ (13/40) or the PROM (12/40) between appointments with 44/80 (55%) keen to avoid between-appointment reporting.

#### Qualitative clinician evaluation

Nine clinicians participated in the pilot with a total of 17 clinician feedback forms submitted (Table 5). Clinicians reported that the Agileware system was *easy* to *neutral* to use, becoming easier with increased familiarity.

**Table 5:** CAPTURE-JIA electronic pilot clinician feedback

1. What went well?	
• Patients seemed enthusiastic about the concept of CAPTURE-JIA data collection. • The Agileware system feels and looks “professional”, flows in accordance with the clinical consultation and becomes easier with familiarity.	
2. What didn’t go so well?	
• Some clinicians reported that the system occasionally crashed mid-data entry. • Forms took much longer to complete for patients who were diagnosed many years earlier (and many of the data items were missing). • Some data items may need a “not known” or “not checked today” tab (e.g. baseline data items, uveitis, height/weight). • Some results may not be available at the time of completing the form (e.g. bloods). How would you advise centres to complete these forms?	
3. What (if anything) would have improved the process?	
• Recruiting new patients only rather than including historical patients.	
4. Additional comments	
• Fantastic to have developed an IT solution to support collection of the dataset. • The forms are far easier to complete fully and less time consuming for newly diagnosed patients. • The forms are considerably less time consuming if completed in retrospect (when all data items are readily available). • May need to consider working with a lead clinician at each centre.	
5. Timings	
• Ranged from 11 to 30 minutes (average 20.7 minutes per form). • Competing priorities may prevent dataset completion in the busy clinical setting. • Data entry became faster with experience.	

### Phase 4

Further to clinician feedback, no changes were made to the consensus-agreed dataset, although a number of minor amendments to the database were implemented. The issue with the system freezing was isolated to one Trust and likely related to firewall restrictions.

The national youth research advisory panel (Your Rheum - https://yourrheum.org) provided additional PPI opinion regarding patient data collection methodologies. Feedback was collated from a virtual Your Rheum meeting involving 6 young people (5F:1M from Northern Ireland (3), Liverpool (1), Manchester (1), Sheffield (1)) and an online survey (*n* = 8). The youngest attendee was 16 years old. Young people felt it important to collect data and appeared surprised that this was not happening already. Anonymity was a high priority. Paper forms were preferable to electronic (*n* = 5/8), although participants did suggest development of a dedicated mobile phone/tablet app (*n* = 4/8), QR code (*n* = 4/8) or direct patient portal (*n* = 4/8) if electronic completion was considered necessary.

A higher proportion of this group indicated that they would like to complete the forms more often in-between clinic appointments (*n* = 5/8). As one young person commented “it’s important to capture ‘a difficult period’ and remember there are good and bad days/weeks”.

## Discussion

This study is the first to demonstrate the feasibility and acceptability of multisite JIA clinical data collection in the UK, using the CAPTURE-JIA dataset and a purpose-built electronic system designed to enable sustainable collection of research-quality patient data in all clinical settings.

The early stages of this phased study identified that paper collection of the CAPTURE-JIA data items is feasible in the routine clinical setting. However, clinicians universally reported paper data collection in parallel to medical notetaking, as time-consuming and non-sustainable. It was felt that a digital tool in the clinical domain, ideally interlocking with local systems, would offer many advantages, including more complete and time-efficient data collection.

As a national healthcare system with no meaningful competition, the NHS should have high quality and widely accessible electronic patient records. However, the creation of accurate and confidential patient records accessible to all healthcare workers has presented a series of significant challenges to the coherent UK-wide digitisation of health. As a result, there is wide between-hospital variation in ERP provider, EPR maturity and EPR capabilities. Limited between-system interoperability and local IT barriers preventing direct data export, presently preclude the collection of a standardised national clinical care dataset in JIA. External vendors, such as Appligo, can be used to implement modern and effective electronic solutions, with the potential to be employed in different ways at different NHS centres, integrating fully with local EPR systems or existing in parallel until full integration becomes possible.

The later phases of the study demonstrated the feasibility and acceptability of a purpose-built electronic solution (Agileware). The solution was easy to use in the clinical setting and the dataset straightforward to store and readily analysable by an experienced academic team. Data entry initially appeared to be time consuming, with clinicians reported completion times ranging from 11 to 30 minutes (average 20.7 minutes per form). However, subsequent focus group discussion highlighted that data entry was significantly faster for new JIA patients; historical clinical data were often very challenging to recall due to challenges with local data collection systems. Some teams reported requiring access to old paper casenotes. This finding is in accordance with our previous work and suggests that future collection of the CAPTURE-JIA dataset should include new patients only, from the point of presentation onwards. This is an important finding and further highlights the pressing need for a feasible and straightforward data collection system, designed to improve local data collection as well as enabling national quality improvement (QI) projects.

Clinicians appear enthusiastic about the concept of electronic data collection, reporting that the Agileware system is intuitive and flows in accordance with the clinical consultation. The trend towards faster data entry over time confirms the clinicians’ comments that the system becomes easier to use with familiarity. Although the system was designed to be used during the clinical consultation, many clinicians reported finding data entry easier and quicker afterwards, once all the results were available. There was a suggestion that centres may benefit from identification of a QI lead to take overall responsibility for ensuring data completion. In response to the challenges precluding historical data completion, there was clear agreement that a future national data collection project should involve new patients only.

CYP/parent pairs participating in the qualitative feedback arm were consistently supportive of the need for national data collection, with many expressing surprise that this is not yet happening. Families were unsure about the need to move away from paper data collection in the clinical environment, with the young people in our small PPI group reporting a preference for paper forms. This is perhaps surprising given the prevalence of smartphone technology amongst CYP and its potential for data capture [21]. We plan further work exploring this finding in more detail across the whole paediatric and adolescent age range.

This study has identified several potential challenges to the success of multicentre data collection include varying EPR maturity, local IT barriers precluding direct data export, limited resources and competing priorities. External data collection systems such as the Agileware system tend to have better functionality than many of the existing hospital EPR systems, allowing local clinicians to make changes to data item definitions or time referent periods. Although the Agileware system can integrate fully with modern EPR systems, local IT barriers precluding direct data export may prevent full integration. In this scenario, parallel external data entry has significant drawbacks, including the additional time required and the potential for missing or incorrect data entries. Data auditing and verification processes would need to be robustly developed. In the short term, parallel external data entry would be a reasonable solution to the current absence of quality of care data but the introduction of modern and fully integrated data collection solutions is a key priority for the NHS and local quality improvement projects exploring challenges and solutions to full integration of the Agileware system are likely to provide important additional information.

Further limitations of the current study relate to the impact of the COVID 19 pandemic on our research. We had initially intended to pilot the Agileware database at six clinical centres across the UK but the much-needed prioritisation of COVID 19 related research resulted in a down-scaling of our original plan.

Implementation of routine electronic collection of the CAPTURE-JIA dataset would improve the completeness of routine clinical data collection. Introduction of a novel mechanism enabling between-centre data analyses would inform clinical practice and service delivery though; (i) identification and setting of standards; (ii) comparison of routine clinical care with agreed standards; (iii) identification and sharing of examples of good practice; (iv) identification and implementation of local and national QI projects. The next step for this project will be a national roll-out of CAPTURE-JIA data collection.

National dataset analyses would be a stepping-stone for much needed quality improvement in paediatric rheumatology across the UK. Our work towards an agreed national dataset for JIA has contextual relevance for the wider paediatric rheumatology community, addressing an area of unmet need and aligning with the Paediatric Global MSK Task Force ‘call to action’ [22]; robust clinical datasets are a powerful way to leverage change through awareness raising about JIA, the benefits of early diagnosis and access to right care, informing health care planners about service provision, workforce planning and staff training. Ultimately the collection of robust datasets will enable meaningful quality improvement projects both nationally and internationally and facilitate improvements in clinical care outcomes for children and young people around the world.

## Conclusions

In conclusion, we have demonstrated the feasibility and acceptability of multicentre CAPTURE-JIA collection, further highlighting the pressing need for national paediatric data collection to drive national quality improvement.

## Supplementary Information

**Additional file 1: Supplementary Material S1.** CAPTURE-JIA Agileware Solution Security Information.

## Data Availability

The datasets used and/or analysed during the current study are available from the corresponding author (FM) on reasonable request.
