# Supplementary material for: Quality improvement in juvenile idiopathic arthritis: a mixed-methods implementation pilot of the CAPTURE-JIA dataset
Source: Pediatr Rheumatol Online J. 2022 Jun 18;20:43. doi: 10.1186/s12969-022-00697-4 (PMC9206126; doi:10.1186/s12969-022-00697-4)
Supplement: Supplementary file 1 — Additional file 1: Supplementary Material S1. CAPTURE-JIA Agileware Solution Security Information. [file 12969_2022_697_MOESM1_ESM.docx]

**Supplementary Material S1: CAPTURE-JIA Agileware Solution Security Information**

**Figure 1: CAPTURE-JIA data collection techniques**

The data collection platform was developed with Appligo using their Agileware platform:

- Agileware is a multi-tenanted Software as a Service (SaaS) solution, hosted in a private cloud.
- Access is over the internet using any modern, HTML5 compatible browser and is not restricted to a specific operating system or device.
- No user generated data is downloaded, stored or cached on the device.
- Each customer is given a personalised sub-domain, which users access via their pre-authorised email address.
- All user access is controlled by local administrators using role-based access control and email authentication.
- In April 2019 Agileware was penetration tested by a third-party facilitator using authenticated, manual testing to imitate a real-world attack.
- Penetration scope included testing features related to potential vulnerabilities in authentication and critical vulnerabilities based on the Open Web Application Security Project (OWASP) Top 10. Report available on request.
- In the instance of the CAPTURE-JIA solution, all patient data is anonymised before being internally shared with the Research Centre (University of Manchester).
- As the data is only “shared” internally, between tenants of the same solution, no data leaves the servers or network.

**Hosting**

- Agileware is hosted by a specialist company, BrightCloud, who have vast experience in the storage and hosting of NHS, patient and other sensitive data.
- The hosted infrastructure is housed in two primary Data Centres based in Park Royal West London and Milton Keynes with offsite backup locations in Maidenhead and Docklands.
- The hosting platform is resilient to single failures in each element and offer a 99.99% uptime guarantee.
- Data security is taken very seriously and as evidence of this BrightCloud are accredited to ISO27001:2013 which is audited by BSi and has a broad scope which covers the business, hosting and management of customer systems.

**Appligo**

- All Appligo employees have completed the Hiscox CyberClear® Academy which is a GCHQ-certified, web-based training platform.
- Appligo is currently undergoing the Cyber Essentials certification as a prerequisite to the Data Protection and Security Toolkit (DPST), provided by NHS Digital. This will be an ongoing piece of work as security standards adapt and evolve to overcome new threats.
- Access to the Agileware databases are restricted to team members with legitimate reason under time specified periods.
- Equipment used to access the databases is stored in key-locked rooms, inside swipe-card accessed buildings with 24/7 security personnel
